# Associations of Indoor Environmental Quality Parameters with Students’ Perceptions in Undergraduate Dormitories: A Field Study in Beijing during a Transition Season

**DOI:** 10.3390/ijerph192416997

**Published:** 2022-12-17

**Authors:** Dan Miao, Xiaodong Cao, Wenxin Zuo

**Affiliations:** School of Aeronautic Science and Engineering, Beihang University, Beijing 100191, China

**Keywords:** dormitory, indoor environmental quality, indoor air quality, noise, carbon dioxide, thermal comfort, well-being

## Abstract

A healthy and comfortable dormitory environment is crucial to the quality of students’ daily lives. In this field study, the indoor environmental quality (IEQ) parameters of undergraduate dormitories in Beijing were measured, while questionnaire surveys were conducted to evaluate the corresponding subjective perceptions of students. Integrated environmental monitoring kits were used to collect temperature, relative humidity, CO_2_, PM_2.5_, PM_10_, TVOC, formaldehyde, and noise data in the investigated dormitories, during the transition season from winter to spring. Questionnaires and scales were distributed to obtain the students’ subjective perceptions of and satisfaction with the IEQ, and their health and well-being status. The measured IEQ data showed that the thermal environment tended to be warm and dry during the heating period. The CO_2_ concentrations seriously exceeded standard levels due to insufficient indoor natural ventilation. Noise exposure could sometimes interfere with students’ rest. The students’ overall satisfaction with the dormitory environment was low, especially in terms of air quality and acoustic environment. The unsatisfactory IEQ factors have led to several health symptoms, poor sleep quality, and slightly lower well-being. Correlations were found between the IEQ parameters and the corresponding subjective perceptions and satisfaction levels. It was speculated that students’ satisfaction and well-being could be effectively improved by appropriately adjusting the corresponding IEQ parameters.

## 1. Introduction

In 2022, the total number of undergraduate and postgraduate students in China could reach nearly 36 million. Dormitories are essential living and resting places for university students, especially at night. As a special indoor environment, a dormitory is characterized by simple furniture, small spaces, a high density of occupants, concentrated activity time, and typically a lack of adjustment measures, which easily create indoor environmental quality (IEQ) problems [1,2]. IEQ has been proven to be closely related to people’s physical and mental health, comfort levels, and study efficiency [3,4]. Therefore, creating a healthy and comfortable dormitory environment is crucial to the quality of students’ daily lives.

Recently, several studies have been conducted on the IEQ issues of student dormitories [1,5,6,7,8,9,10,11,12,13]. Several studies have focused on the relationship between the ventilation rate and students’ health status, as well as on the indoor air quality (IAQ) of dormitories. Yang et al. [1] investigated ventilation and air quality in dormitories during the summer in Nanjing, China. The measured I/O ratios of PM_2.5_ and ozone were in the range from 0.42 to 0.79 and from 0.21 to 1.00, respectively. The authors recommended the use of open/closed window strategies to reduce indoor pollutant levels. Liu et al. [7] evaluated the ventilation rates of dormitories during the heating season. They found that low ventilation rates could affect students’ sleep quality. As investigated by Yang et al. [8], crowded dormitories and low ventilation rates were associated with more common colds and influenza infections among college students. Thermal comfort is also a focus of the research on dormitory IEQ. Wang et al. [9] conducted a field study in university classrooms and dormitories during the heating period in Harbin, China. The authors found that students felt more thermally comfortable in dormitories due to thermal adaptation and less clothing insulation. Sun et al. [10] investigated the thermal comfort of student apartments in cold areas using both field tests and questionnaires. The authors proposed heating-system adjustment strategies based on the target thermal comfort temperature for different periods, through the analysis of student behavior and historical temperature. A field study of campus dormitories in Taiwan [11] showed that the operative temperatures of thermal neutrality and thermal preference were 25.4 °C and 24.8 °C, respectively. Zhang et al. [12] monitored the IEQ of dormitories during the winter in Shanghai, China, where there was no central heating equipment in the winter. Students had to close windows to reduce heat loss, which resulted in elevated indoor CO_2_ concentrations. The authors recommended providing central heating systems or individual heating units in dormitory buildings in the Yangtze Delta region. As summarized, most relevant studies focused on investigating either the thermal comfort or IAQ of dormitories. However, comprehensive analyses of multiple IEQ parameters and corresponding subjective perceptions are still lacking. Therefore, further studies are still needed to better understand the impact of the overall IEQ of dormitories on students’ perceptions and well-being.

Local climatic conditions could affect the IEQ performance to a certain extent. The weather in Beijing is typically cold and dry during the transition season from winter to spring, and the indoor environmental adjustment of dormitories basically relies on both central heating and natural ventilation. However, students rarely open windows for ventilation due to large indoor/outdoor temperature differences. The decreased fresh air introduced into dormitories may lead to deteriorating IAQ. We were also interested in observing the change in the thermal environment during and after the central heating period. Hence, we conducted a field investigation of objective IEQ conditions and students’ perceptions of undergraduate dormitories in Beijing, China during a transition season. Indoor environmental parameters were monitored during this one-month pilot study. Meanwhile, subjective questionnaires and scales were distributed to obtain students’ perceptions of and satisfaction with the dormitory environment, as well as their health and well-being conditions. The associations of the IEQ parameters with corresponding students’ perceptions and satisfaction were then analyzed.

## 2. Methods

### 2.1. Measurement of IEQ Parameters

**Figure 1:**
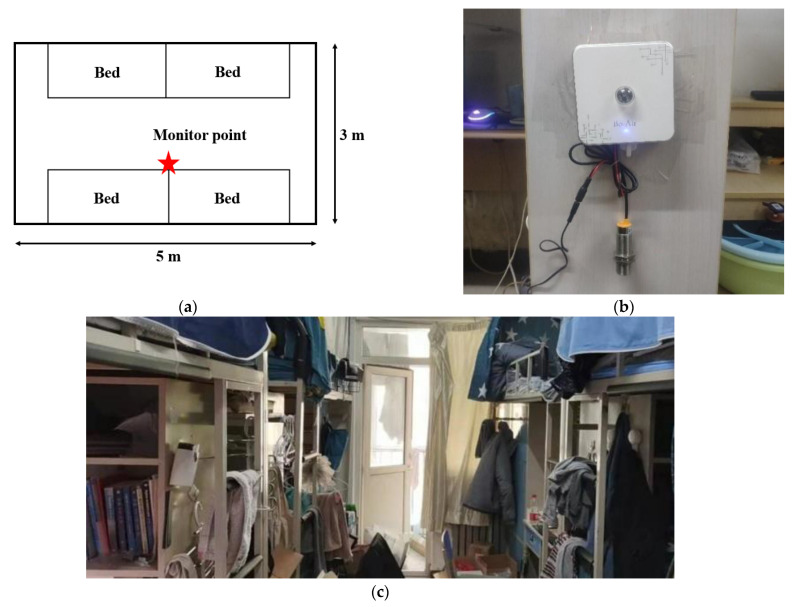
(**a**) Four-bed dormitory room plan; (**b**) Installation location of the monitoring kit; (**c**) Typical scene in a dormitory.

The field study was carried out in two four-bed male undergraduate dormitories (see Figure 1) in Beijing from 12 March 2022 to 9 April 2022. The central-heating period was stopped on March 22. The two dormitories are located on the 5th and 7th floors of a residential building. The objective IEQ measurement was performed with multiparameter online IEQ monitoring kits (BOAIR-C9W, Shenzhen, China), which were calibrated by the manufacturer. The monitoring kits were also calibrated against more comprehensive instruments such as TSI 8530 and testo 440, before the formal measurement. The measured IEQ parameters included temperature, relative humidity (RH), concentrations of CO_2_, PM_2.5_, PM_10_, TVOC and formaldehyde, and the A-weighted sound pressure level (SPL) of noise. The measurement resolution and accuracy of these parameters are shown in Table 1. According to the Chinese national standard for indoor air quality [14], 1~3 sampling points need to be set up for a room with an area of less than 50 m^2^. Considering that the area of dormitory is about 15 m^2^, only one sampling point is required. The height of the sampling point should be consistent with the height of the human respiratory zone. Students basically sat on chairs during the measurement, so the sampling height was determined to be 1 m. Therefore, one monitoring kit was installed at a position near the geometric center of each dormitory at a height of 1 m from the ground, as shown in Figure 1b. The IEQ parameters were monitored for one hour (from 23:00 to 0:00) per day for 29 consecutive days with a sampling interval of 1 min. This measurement time period was chosen because all students had been active in their dorms for a while and were ready to sleep during this period. Thus, the measured IEQ conditions were relatively stable and representative.

The monitored IEQ data were further compared against the relevant standard values. According to the Chinese national standard for indoor air quality [14], the permissible range or limit values of the thermal and IAQ parameters are shown in Table 2. The night-time equivalent sound pressure level limit is 45 dB(A) in residential buildings, as required by the Chinese environmental quality standard for noise (GB 3096-2008) [15].

### 2.2. Subjective Survey

Subjective surveys were conducted to measure the students’ perceptions of and satisfaction with IEQ and their health and well-being status. The survey mainly consisted of the following questionnaires and scales: the (1) Subjective evaluation scale for IEQ factors; (2) Health symptom scale; (3) Sleep quality questionnaire; (4) Positive and Negative Affect Schedule (PANAS); and (5) WHO-5 well-being index scale. These questionnaires and scales were distributed to eight students in the measured dormitories, seven times throughout the measurement. Students were asked to finish the survey within the IEQ measurement period. The survey dates were selected primarily considering the coverage of a wider range of the IEQ parameters, both during and after the heating period.

The subjective evaluation scale for the IEQ was used to investigate students’ environmental perceptions of and satisfaction with temperature, humidity, air quality and noise. The perception and satisfaction of the IEQ factors used a 7-point scale, with corresponding options summarized in Table 3. The purpose of the health symptom scale was to investigate the extent of sick-building syndrome using a 5-point scale with the following options: not at all (1), a little (2), somewhat (3), quite (4), and very much (5). The sleep quality questionnaire was used to evaluate the sleep quality of students and the main factors that could interfere with sleep.

The PANAS [16] is a standardized tool for measuring changes in human sentiment. The PANAS comprises two 10-item scales measuring both positive and negative emotions, with a 5-point scale of 1 (not at all) to 5 (very much) used for each item. The positive emotion score and negative emotion score were summed separately to create a total score range from 10 to 50. The higher the total score, the higher the corresponding positive or negative sentiment level. The WHO-5 well-being index scale is a five-question scale, which is commonly used to measure the subjective well-being of respondents [17]. The well-being index of each question used a 6-point scale with the following options: never (0), sometimes (1), less than half the time (2), more than half the time (3), most of the time (4), and all of the time (5). Higher total well-being index scores represent better subjectively rated well-being. A total score of below 13 indicates poor quality of life and is an indication for the further evaluation of depression.

## 3. Results

### 3.1. Measured IEQ Parameters

#### 3.1.1. Thermal Environmental Parameters

**Figure 2:**
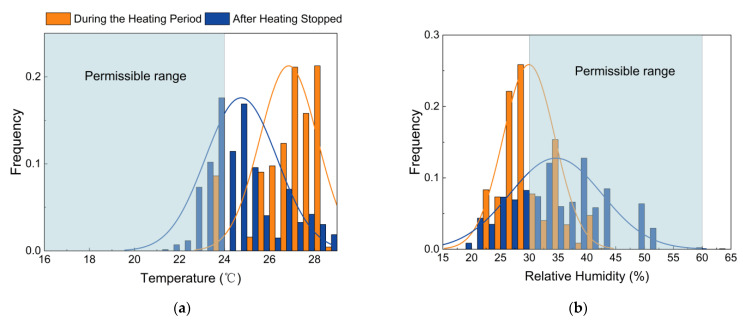
Histograms of thermal environmental parameters: (**a**) temperature; (**b**) RH.

Figure 2a shows the frequency distributions of indoor temperature data. The fitting curves in the histograms represent the normal distributions’ fit corresponding to the mean and standard deviation of the data. During the heating period, indoor temperature mainly fluctuated from 26 °C to 28 °C. The indoor temperature was significantly higher than the thermal comfort temperature ranging from 16 °C to 24 °C, only 8.6% of which met the requirement range. After the heating stopped, the indoor temperature was concentrated between 23.5 °C and 25.5 °C, 37% of which fell within the requirement range. It was apparent that the indoor temperature during the heating period was approximately 2~3 °C higher than that after heating stopped. As shown in Figure 2b, the RH ranged from 23.2% to 42.7% during the heating period, 36% of which met the requirement range. After heating stopped, the RH mostly fluctuated from 30% to 45%, 68.5% of which met the comfort range. The results indicated that the indoor environment after heating was more humid than that during the heating period. In sum, the indoor thermal environment tended to be warm and dry during the heating period but became more thermally comfortable after the central heating stopped.

#### 3.1.2. IAQ Parameters

**Figure 3:**
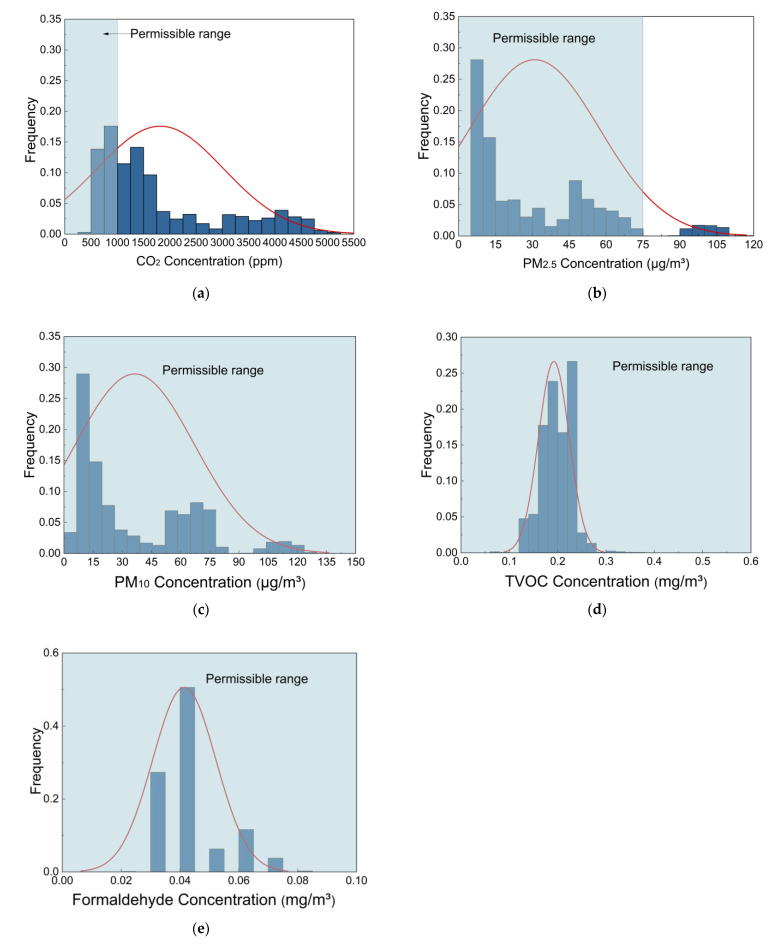
Histograms of IAQ parameters: (**a**) CO_2_; (**b**) PM_2.5_; (**c**) PM_10_; (**d**) TVOC; (**e**) formaldehyde.

Figure 3 presents the indoor concentration distributions of CO_2_, PM_2.5_, PM_10_, TVOC, and formaldehyde during the measurement period. As shown in Figure 3a, 68% of CO_2_ concentrations exceeded the permissible exposure limit of 1000 ppm. In addition, the average indoor CO_2_ concentration was only lower than the limit value on seven days, and it could even accumulate to higher than 4000 ppm. The high levels of CO_2_ have indicated that the indoor natural ventilation was inadequate, due to the fact that windows were rarely opened at night. Figure 3b shows that the PM_2.5_ concentrations ranged from 5 µg/m^3^ to 115 µg/m^3^. Only 7% of the PM_2.5_ concentrations exceeded the limit value of 75 µg/m^3^. As shown in Figure 3c, the PM_10_ concentrations ranged from 6 µg/m^3^ to 129 µg/m^3^, all falling below the limit value of 150 µg/m^3^. Overall, the PM concentrations basically met the requirements of the Chinese national standard.

As revealed by Figure 3d, the TVOC concentrations mainly ranged from 0.13 mg/m^3^ to 0.25 mg/m^3^, with the highest value reaching 0.36 mg/m^3^, which was lower than the limit value of 0.6 mg/m^3^. Figure 3e shows that the measured formaldehyde concentrations were below the limit value of 0.1 mg/m^3^. The results indicated that the measured TVOC (including formaldehyde) concentrations were compliant with the Chinese national standard. Thus, the VOCs emitted by densely arranged furniture and decorative paint in the dormitories were basically at acceptable levels.

#### 3.1.3. Noise Sound Pressure Level

**Figure 4:**
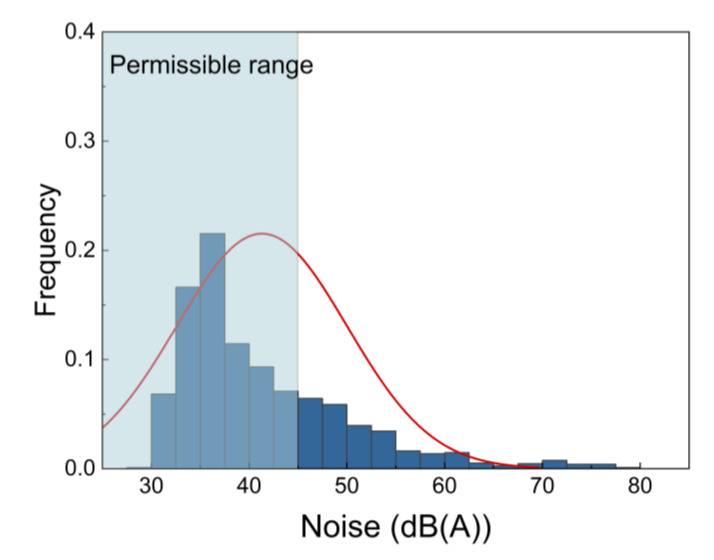
Histograms of noise SPL.

As shown in Figure 4, the measured SPL ranged from 26.9 dB(A) to 79.8 dB(A), with 28% exceeding the night-time equivalent limit value of 45 dB(A) [15]. Excessive noise likely resulted from personal entertainment audio systems and loud conversations between students, which may interfere with students’ rest and subsequent sleep quality.

### 3.2. Environmental Perceptions and Satisfaction

#### 3.2.1. Thermal Environmental Parameters

**Figure 5:**
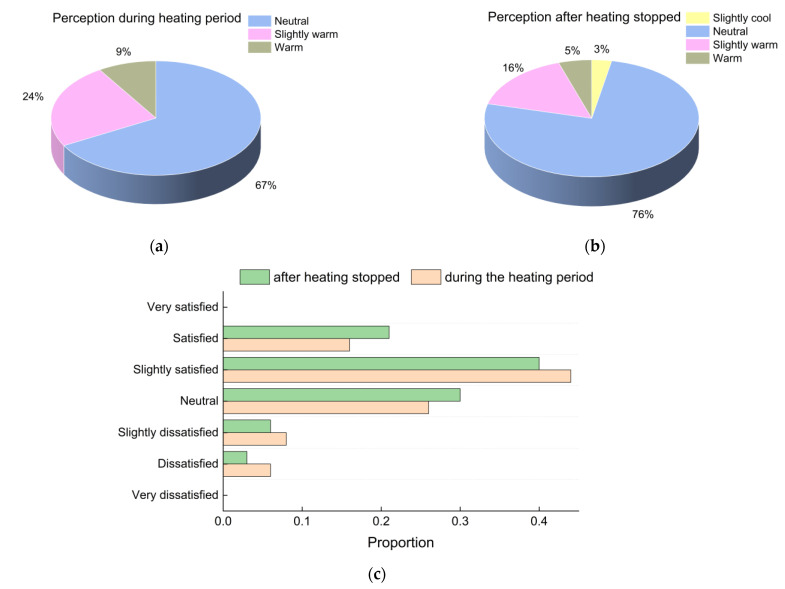
Subjective evaluation of temperature: (**a**) perception during the heating period; (**b**) perception after heating stopped; (**c**) satisfaction proportions.

The measured thermal environmental data were significantly different during and after heating stopped. As shown in Figure 5, 33% of the students felt warm during the heating period, but the proportion of students who felt warm dropped to 21% after the heating stopped. Furthermore, satisfaction with the thermal environment also improved after the heating stopped, indicating that a larger portion of students perceived the indoor air temperature to be more comfortable.

**Figure 6:**
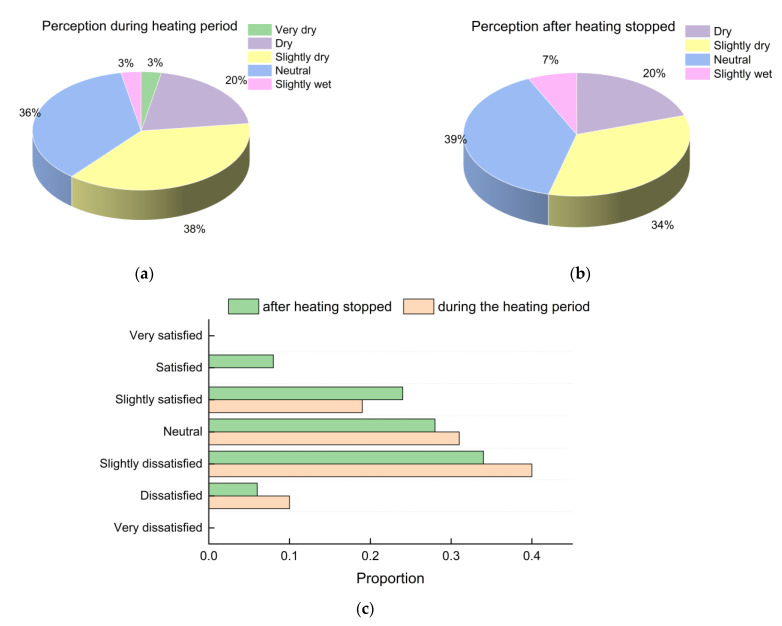
Subjective evaluation of RH: (**a**) perception during the heating period; (**b**) perception after heating stopped; (**c**) satisfaction proportions.

According to the measured RH data, central heating also had an effect on the indoor humidity levels. As shown in Figure 6, during the heating period, 61% of the students felt that the environment was dry, including 3% of them feeling very dry. However, after the heating stopped, the proportion of students feeling that the environment was dry decreased to 54%, while satisfaction with the humidity environment increased from 19% to 32%. In sum, the students’ satisfaction with humidity levels also improved somewhat after the central heating stopped.

#### 3.2.2. IAQ

**Figure 7:**
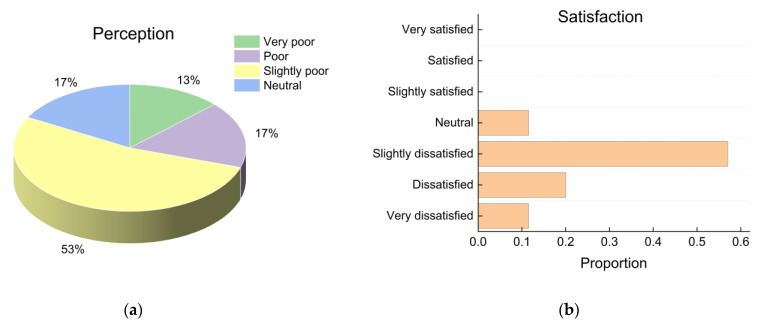
Subjective evaluation of IAQ: (**a**) perception; (**b**) satisfaction proportions.

Figure 7 shows the proportions of subjective perceptions of and satisfaction with the IAQ. Eighty-three percent of the students perceived the IAQ to be poor, and 89% of the students were not satisfied with the IAQ during the measurement period. No students were satisfied with IAQ conditions. The subjective evaluation results for the IAQ corresponded with the measured CO_2_ concentrations, which far exceeded the standard limit values. The poor ventilation performance could be attributed to seldomly opened windows due to low outdoor temperatures.

#### 3.2.3. Acoustic Environment

**Figure 8:**
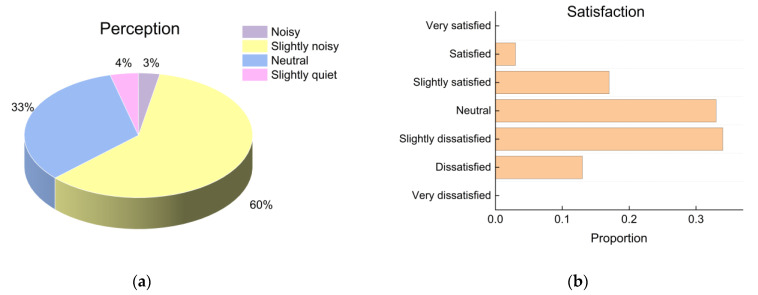
Subjective evaluation of noise: (**a**) perception; (**b**) satisfaction proportions.

Figure 8 shows that 63% of the students felt that the acoustic environment of the dormitory was noisy. At night, the source of noise was mainly the conversations and entertainment inside the dormitory. Furthermore, nearly half of the students were dissatisfied with the acoustic environment. The results indicated that even moderate over-exposure to noise at night could lead to a relatively high level of dissatisfaction among students.

#### 3.2.4. Overall Satisfaction

**Figure 9:**
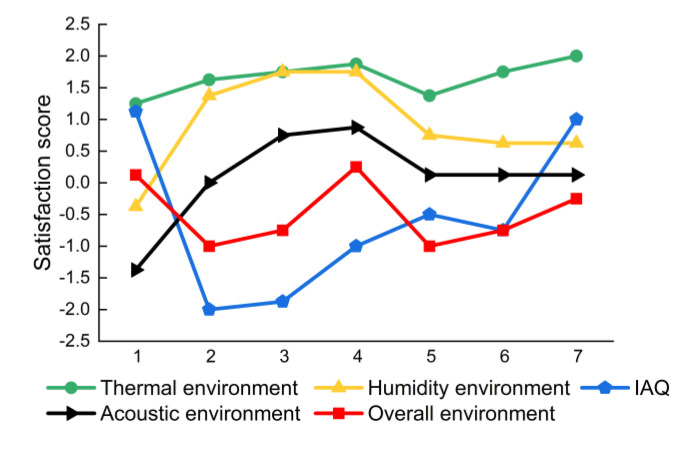
Comparison between single and overall IEQ satisfaction scores in the seven subjective surveys.

In sum, a relatively large proportion of students was dissatisfied with the IAQ and the acoustic environment. However, there was a lack of appropriate adjustment measures to improve the corresponding IEQ factors. At the same time, students may be dissatisfied with the confined layout and simple furniture of dormitories. Therefore, 81% of the students were dissatisfied with the overall IEQ of the dormitories. The Spearman’s correlation coefficients for ranked data were used to examine the relationships between the single and overall IEQ satisfaction scores. The correlation analysis was performed using the SPSS 25 (IBM Corp.: Armonk, NY, USA). As shown in Figure 9, the variation trends of the IAQ and thermal environment satisfaction scores were generally related to the overall IEQ satisfaction scores with correlation coefficients of 0.4 and 0.367, respectively. The comparative results indicated that the IAQ and the thermal environment could be principal influencing factors for overall satisfaction in the investigated dormitories. The results were consistent with the call of Wei et al. [18] to prioritize the IAQ for the proper characterization of the IEQ in buildings, followed by thermal, lighting and acoustic conditions. Moreover, Tang et al. [19] compared different models to evaluate the overall IEQ satisfaction. They found that unsatisfactory factors often had a dominant negative impact on occupants’ perceptions, which cannot be counteracted by higher satisfaction with other factors. This helps explain why the overall satisfaction scores were basically lower than the corresponding satisfaction scores for thermal, humidity and acoustic conditions. Given this perspective, improvements to the most dissatisfying factor (e.g., IAQ in this study) could be significantly effective in increasing the overall IEQ satisfaction.

### 3.3. Perceived Health and Well-Being

#### 3.3.1. Health Symptoms

**Figure 10:**
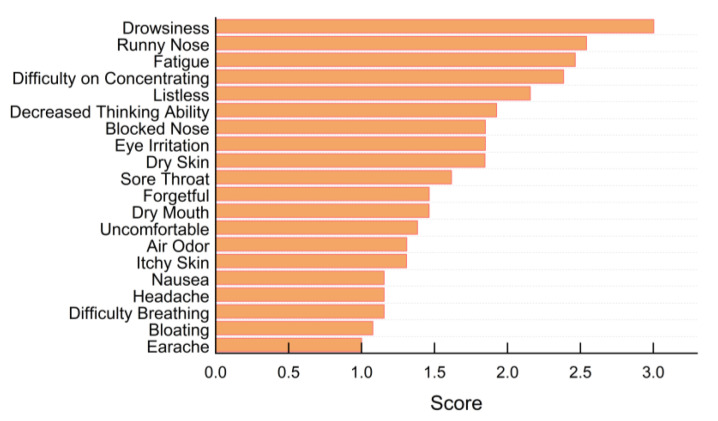
Average score distributions of health symptoms.

The average scores of different acute health symptoms are sorted in Figure 10. In general, health symptoms of relatively higher frequency (score higher than 2) include drowsiness, a runny nose, fatigue, difficulty concentrating, listlessness, and decreased thinking ability. In addition, almost every symptom except earache was reported by the students. The results indicated that the unsatisfactory IEQ factors could have led to adverse effects on students’ health status, particularly on cognitive performance.

#### 3.3.2. Well-Being

**Figure 11:**
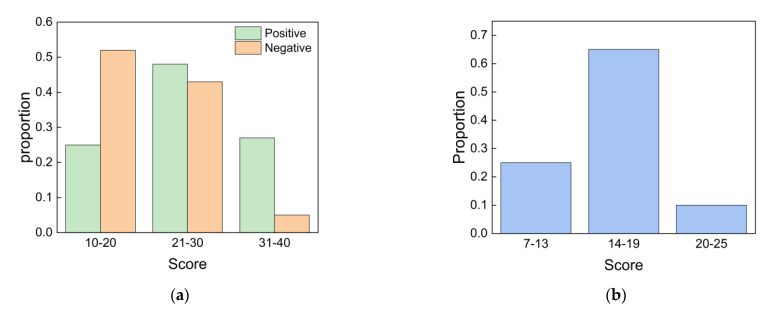
Subjective evaluation of well-being: (**a**) PANAS score; (**b**) WHO-5 well-being index score.

The students’ well-being was assessed using both the PANAS and the WHO-5 well-being index scale. As shown in Figure 11a, the average scores of positive and negative emotions were 25.2 and 18.7, respectively. More than 20% of the students’ positive emotion scores were in the relatively low range from 10 to 20. In general, the positive emotions of students were slightly stronger than their negative emotions, and both were in a moderate range. As shown in Figure 11b, the average well-being score was 16.8, but more than 20% of students scored below 13. This result indicated that the overall well-being index was only slightly higher than the minimum standard. On the whole, the students’ well-being was average, which may be affected by unsatisfactory IEQ to a certain extent.

**Figure 12:**
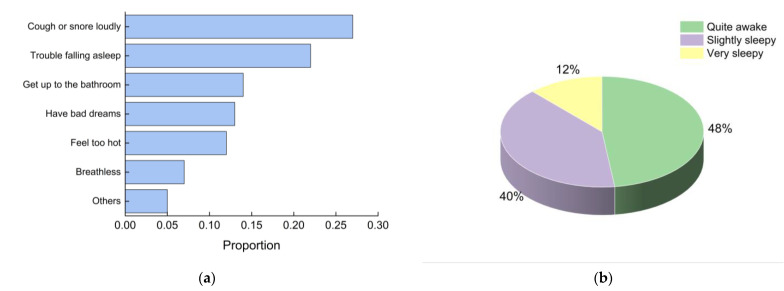
Subjective evaluation of sleep quality: (**a**) factors affecting sleep quality; (**b**) state upon waking up.

According to the results of the sleep quality questionnaires, most of the students fell asleep late, and some even fell asleep at 3:30 a.m. at the latest. Their sleep time was relatively short at 6 h on average. More than half of the students indicated that their sleep quality was poor. The distributions of factors affecting sleep quality are shown in Figure 12a, mainly including coughing or snoring loudly and trouble falling asleep. Feeling too hot and breathless were also among the factors that led to poor sleep quality. Figure 12b shows that 52% of the students still felt drowsy upon waking up. The results suggested that unsatisfactory IEQ at night could negatively affect sleep quality.

## 4. Discussion

### 4.1. Associations of IEQ Parameters with Students’ Perceptions

**Figure 13:**
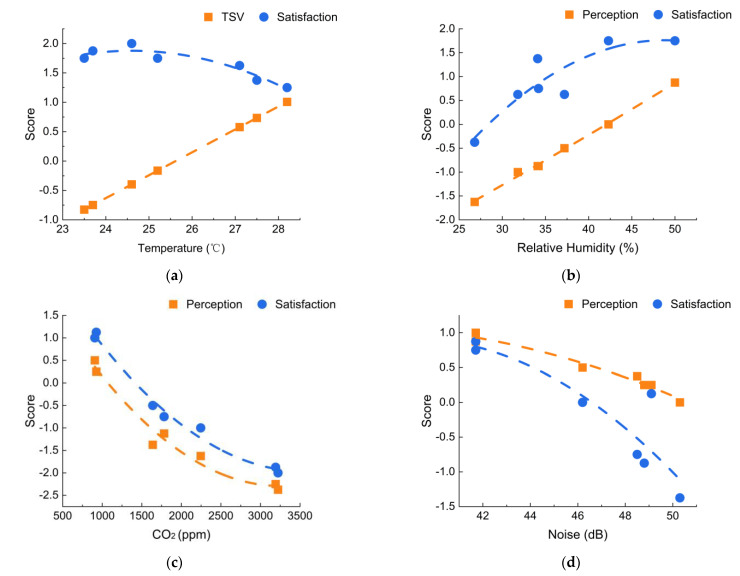
Associations between (**a**) temperature; (**b**) RH; (**c**) CO_2_; (**d**) noise and corresponding environmental perception and satisfaction scores.

As revealed by Figure 13a, the TSV had a clear positive linear relationship with temperature. According to the regression results, thermal sensation was neutral (TSV = 0) when the temperature was 25.6 °C. The relationship between temperature and the thermal environment satisfaction score followed a quadratic function curve [20]. The regression curve indicated that the thermal environment satisfaction reached the maximum when the temperature was 24.7 °C. The thermal satisfaction decreased whether the temperature rose or fell. This indicated that the students were most satisfied with the thermal environment when the temperature was slightly lower than the thermally neutral state. The associations between the RH and the humidity perception and satisfaction scores are shown in Figure 13b. Similar to the TSV, the humidity perception score showed a positive quasilinear relationship with the RH. Humidity perceptions were predicted to be in neutral when the RH was equal to 42.3%. The relationship between the RH and the humidity satisfaction score approximately followed a quadratic function curve. The humidity satisfaction tended to reach the highest level when the RH was equal to 48.5%, which was slightly higher than the RH value in the neutral state.

The indoor CO_2_ concentration is typically considered to be a proxy indicator of ventilation and the IAQ [18]. As shown in Figure 13c, the curve associations of the IAQ perception and satisfaction scores versus the CO_2_ concentration were quite similar. The IAQ perception and satisfaction scores began to decrease sharply when the CO_2_ concentration exceeded 900 ppm. The students’ perception of and satisfaction with the IAQ continued to decrease with the elevated CO_2_ concentrations of above 1500 ppm but with a gradually decreasing change rate. As shown in Figure 13d, the noise SPL was negatively correlated with the acoustic perception and satisfaction scores. As the SPL increased, the students’ acoustic perception and satisfaction scores decreased significantly. According to the regression curves, the acoustic perception and satisfaction scores began to drop more rapidly when the noise sound pressure level exceeded 45 dB(A), and the satisfaction score decreased significantly faster. This indicated that excessive noise could seriously reduce the students’ satisfaction with the acoustic environment.

**Figure 14:**
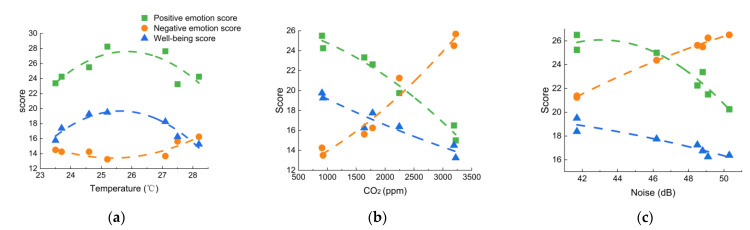
Associations of (**a**) temperature, (**b**) CO_2_, and (**c**) noise with emotion and well-being index scores.

Relevant studies have shown that indoor environmental factors could have varying impacts on human psychology [21,22,23]. As shown in Figure 14a, when the temperature was too high or low, the positive emotion and well-being index scores decreased, while the negative emotion score increased. Figure 14b shows that as the CO_2_ concentration increased, the positive emotion and well-being index scores decreased significantly, while the negative emotion scores increased rapidly. Furthermore, the noise exposure had similar effects on students’ emotions and well-being. As shown in Figure 14c, the students’ positive emotion and well-being index scores gradually decreased, while their negative emotion scores increased with the elevated SPL values. Specifically, the positive emotion scores declined faster when the SPL exceeded 46.2 dB(A).

In sum, the deteriorated IEQ parameters are correlated with a decline in subjective perception and satisfaction levels, as well as with lower sentiment and well-being states. Therefore, it is necessary to take effective adjustment measures to improve the IEQ conditions to create healthy and comfortable dormitory environments.

### 4.2. Measures for Improving IEQ

Based on the above analyses, it could be inferred that students’ feelings and well-being could be enhanced by feasible improvement measures for the corresponding IEQ parameters. In this study, the average indoor CO_2_ concentration was 1774 ppm, and the dissatisfaction rate of the IAQ was nearly 90% during the measurement period. Similarly, a recent field study indicated that the CO_2_ concentrations were higher than 1000 ppm in 90% of the investigated dormitories in Nanjing, China [13]. Higher indoor CO_2_ and PM_2.5_ levels were identified to be associated with decreased cognitive function in a multicountry longitudinal observational study [24]. Improvements in the IAQ through higher ventilation rates were also associated with a reduced incidence rate of health symptoms among undergraduate students [25]. It is generally agreed that opening windows for ventilation is an effective and economical means to improve the IAQ, by introducing fresh outdoor air and reducing indoor CO_2_ and other pollutant concentrations [26]. However, it is worth noting that opening windows may also introduce atmospheric pollutants, depending on how polluted the outdoor air is. Therefore, relevant data from the meteorological station closest to the field site were used to further investigate outdoor air quality conditions.

**Figure 15:**
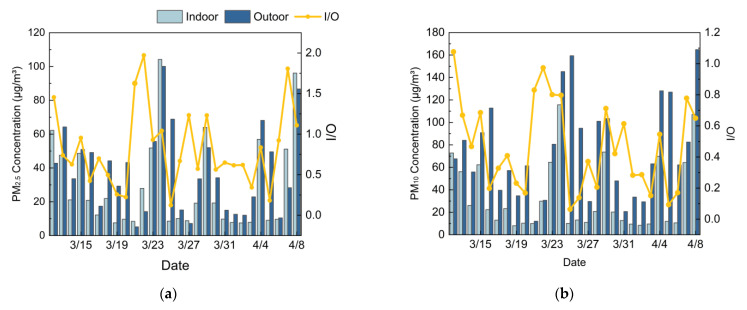
Comparison between indoor and outdoor PM concentrations: (**a**) PM_2.5_; (**b**) PM_10_.

Figure 15 shows the indoor and outdoor (I/O) ratios of the PM_2.5_ and PM_10_ concentrations during the measurement period. As shown in Figure 15a, the I/O ratios of PM_2.5_ ranged from 0.12 to 1.97, with the indoor concentrations exceeding the outdoor concentrations on eight days. Figure 15b shows that the I/O ratios of the PM_10_ ranged from 0.06 to 1.08, with only one day of higher indoor PM_10_ concentrations recorded. Moreover, the indoor and outdoor PM_2.5_ and PM_10_ concentrations were significantly correlated (*p* < 0.01), with the correlation coefficients of 0.647 and 0.554, respectively. It can be inferred that the indoor PM concentrations were normally lower than the outdoor concentrations and fluctuated with the corresponding outdoor concentrations. The air quality index (AQI) [27] quantitatively describes the outdoor air pollution conditions based on the monitored concentrations of SO_2_, NO_2_, PM_10_, PM_2.5_, O_3_ and CO. The AQI measures six pollution levels: excellent (0–50), good (51–100), light pollution (101–150), moderate pollution (151–200), heavy pollution (201–300), and serious pollution (>300) [28]. During the measurement period, there was only one day of heavy outdoor air pollution, with an AQI of 205. The outdoor air quality was good or excellent on 87% of the measured days, with an average the AQI of 70. Therefore, it is feasible to improve the IAQ of dormitories by properly opening windows for natural ventilation. However, when the outdoor air pollution is severe, natural ventilation should be restricted to prevent the indoor concentration of the PM or other air pollutants from exceeding the allowable exposure limit. In addition, portable air purifiers [29,30] can also be used to reduce indoor PM exposure, when the outdoor PM concentration is high.

In our study, the average temperatures during and after the central heating period were 27.0 °C and 24.1 °C, respectively. In similar studies [9,12,13], the average temperatures of the dormitories measured in Harbin, Nanjing, and Shanghai during the winter were 23 °C, 15.8 °C, and 12.1 °C, respectively. The measured indoor temperatures were significantly lower in Shanghai and Nanjing, due to the lack of central heating systems. The students’ thermal satisfaction is predicted to be the highest when the indoor temperature is approximately 24.7 °C (Figure 13). Thus, when the indoor temperature is relatively low after the heating is stopped, personal heaters [31,32] can be used to improve the thermal condition of dormitory environment. Meanwhile, opening windows for ventilation may not only improve the IAQ, but also help lower the slightly higher indoor temperature during the heating season.

In this study, the average RH during and after the central heating period were 29.9% and 34.6%, respectively. The average RH during the whole measurement period was 33.8%, which was just above the minimum standard value of 30%. Dissatisfaction with humidity levels even reached 81% during the heating period. In previous studies [9,12,13], the average RH of the dormitories in Harbin, Nanjing, and Shanghai during the winter was measured as 36.7%, 50.9% and 69.8%, respectively. It is evident that the indoor RH in Beijing and Harbin (northern China) is significantly lower than that in southern Chinese cities, due to the relatively dry outdoor environment during the winter. Using a portable ultrasonic humidifier could be an affordable measure to improve indoor humidity levels in the dorms [33]. In addition, attention should be given to the reduction in excessive noise, especially at night. Applicable noise reduction measures [34] include wearing earphones for personal entertainment, engaging in quiet conversation, using thicker curtains, and sound masking with light music, etc.

## 5. Conclusions

Integrating objective measurements and subjective surveys, this study preliminarily explored the associations of the dormitory IEQ parameters with students’ perceptions in Beijing during the transition season from winter to spring. The main findings are summarized as follows:

(1) The thermal environment was relatively warm during the heating period, and temperatures dropped after the central heating stopped. The RH was generally moderate after the heating stopped, but there was some dryness during the heating period. The CO_2_ concentrations seriously exceeded the limit value, indicating insufficient natural ventilation. The indoor PM concentrations were basically below the limit values. The variations in the indoor PM concentrations had a relatively strong correlation with the corresponding outdoor concentrations. The noise sound pressure level sometimes exceeded 45 dB(A), which may seriously affect students’ rest and sleep.
(2) The perceived thermal environment tended to be warm and dry during the heating period. The students thought that the thermal environment had become more comfortable after the central heating stopped, and their satisfaction with the temperature and the RH also increased. The overall satisfaction with the dormitory environment was quite low, especially for the air quality and acoustic conditions. The IEQ problems probably had a negative impact on students’ health and well-being. The students reported that many experienced acute health symptoms, as well as slightly low well-being. Sleep quality could also be partially affected by poor IEQ conditions.
(3) Subjective perceptions and satisfaction were found to be well associated with the corresponding IEQ parameters. It could be speculated that students’ satisfaction and well-being could be effectively improved by reasonably adjusting the corresponding environmental parameters. Hence, feasible measures such as properly opening windows for ventilation, using heaters and humidifiers, and wearing earphones for personal entertainment are necessary to improve the IEQ of dormitories.

Nevertheless, the generalization of these pilot study results should be limited to cases based on similar research contexts and climatic conditions. The present findings are less robust due to the small sample size and short research period examined. Based on the proposed research methodology, further studies can be conducted on a more extensive range of dormitories, student respondents and measurement periods. More cognitive tests and wearable physiological measurements should also be included in field work. Then, the comprehensive effects of multiple IEQ parameters on subjective perceptions, physiological responses, and cognitive performance should be better revealed thorough big data model analyses.

## Figures and Tables

**Table 1:** Specifications of the environmental monitoring kit.

Category	Range	Resolution	Accuracy
Temperature	−40~80 °C	0.1 °C	±0.5 °C
RH	0~100% RH	0.1% RH	±5% RH
CO_2_	0~5000 ppm	1 ppm	±40 ppm ± 3% reading
PM_2.5_	0~1000 µg/m^3^	1 µg/m^3^	±10% F. S. ^1^
PM_10_	0~2000 µg/m^3^	1 µg/m^3^	±10% F. S.
TVOC	0~5.00 mg/m^3^	0.01 mg/m^3^	<±20%
Formaldehyde	0~200 ppm	0.01 ppm	<±0.05 ppm
Sound pressure level	35~120 dB(A)	0.1 dB(A)	1.5 dB(A)

^1^ F. S.: Full scale.

**Table 2:** Permissible values of thermal and IAQ parameters according to the Chinese national standard for indoor air quality [14].

IEQ Parameters	Permissible Values	Notes
Temperature	16~24 °C	Winter
RH	30~60%	Winter
CO_2_	1000 ppm	24 h average
PM_2.5_	0.075 mg/m^3^	24 h average
PM_10_	0.15 mg/m^3^	24 h average
Formaldehyde	0.1 mg/m^3^	1 h average
TVOC	0.6 mg/m^3^	8 h average

**Table 3:** Options of subjective evaluation scale for IEQ factors.

Scale Score	Thermal Environment	Humidity Environment	Acoustic Environment	IAQ	Satisfaction Level
−3	cold	very dry	very noisy	very poor	very dissatisfied
−2	cool	dry	noisy	poor	dissatisfied
−1	slightly cool	slightly dry	slightly noisy	slightly poor	slightly dissatisfied
0	neutral	neutral	neutral	neutral	neutral
1	slightly warm	slightly humid	slightly quiet	slightly good	slightly satisfied
2	warm	humid	quiet	good	satisfied
3	hot	very humid	very quiet	very good	very satisfied

## Data Availability

The data presented in this study are available upon request from the corresponding author.
